# [Corrigendum] miR‑137 suppresses proliferation, migration and invasion of colon cancer cell lines by targeting TCF4

**DOI:** 10.3892/ol.2024.14378

**Published:** 2024-04-03

**Authors:** Wei-Ping Bi, Min Xia, Xin-Jian Wang

Oncol Lett 15: 8744–8748, 2018; DOI: 10.3892/ol.2018.8364

Following the publication of the above article, the authors contacted the Editorial Office to inform us that several errors were made in the compilation of the data panels shown for the cell migration and invasion assay experiments in Fig. 3, as it appeared on p. 8747. The Editorial Office then conducted its own independent investigation of the matter, and granted the authors permission to publish a corrigendum. Subsequently, it was noted that the western blots shown in Fig. 4 on the same page appeared to contain strikingly similar control β-actin blots, even though the blots were intended to show the results from differently performed experiments.

The revised and corrected versions of Figs. 3 and 4 are shown on the next page. All the authors approve of the publication of this corrigendum, and the authors are grateful to the Editor of *Oncology Letters* for granting them the opportunity to publish this. The authors also apologize to the readership for any inconvenience caused.

## Figures and Tables

**Figure 3.**
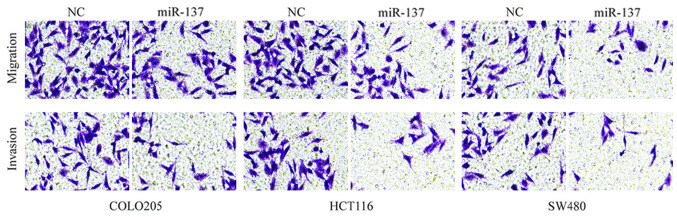
miR-137 suppresses migration and invasion in COLO205, HCT116 and SW480 colon cancer cell lines following transfection with miR-137. Representative images of migratory and invasive cells (magnification, ×100) are shown. Cell migration and invasiveness was significantly decreased in miR-137-transfected cells with respect to the corresponding NC. NC, negative control; miR-137, microRNA-137.

**Figure 4.**
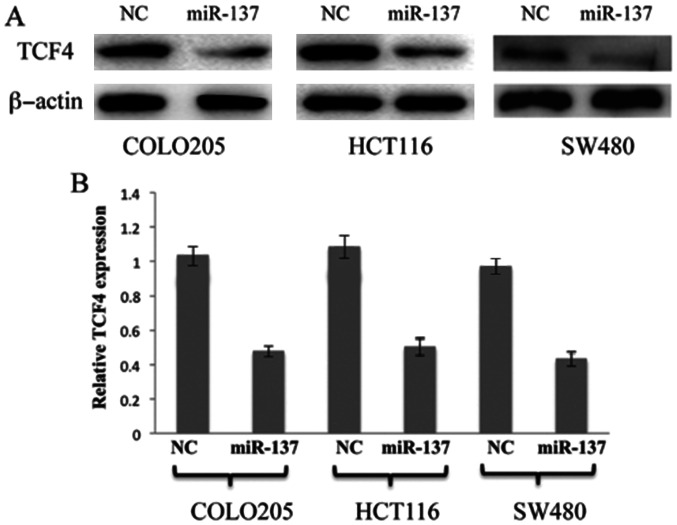
miR-137 negatively regulates TCF4 expression in colon cancer COLO205, HCT116 and SW480 cell lines. (A) Western blot analysis of TCF4 protein in colon cancer COLO205, HCT116 and SW480 cell lines after overexpression of miR150. Corresponding NCs and additional control (β-actin) were used in all the experiments. (B) Reverse transcription-quantitative polymerase chain reaction analysis of TCF4 mRNA in colon cancer COLO205, HCT116 and SW480 cell lines following overexpression of miR150. Bars indicate the standard deviation. NC, negative control; miR-137, microRNA-137; TCF, transcription factor.

